# 3D reconstruction of small bowel lesions using stereo camera-based capsule endoscopy

**DOI:** 10.1038/s41598-020-62935-7

**Published:** 2020-04-07

**Authors:** Seung-Joo Nam, Yun Jeong Lim, Ji Hyung Nam, Hyun Seok Lee, Youngbae Hwang, Junseok Park, Hoon Jai Chun

**Affiliations:** 1 Department of Internal Medicine, Kangwon National University School of Medicine, Chuncheon, Korea; 2 Department of Internal Medicine, Dongguk University College of Medicine, Dongguk University Ilsan Hospital, Goyang, Korea; 3 Department of Internal Medicine, School of Medicine, Kyungpook National University, Kyungpook National University Hospital, Daegu, Korea; 4 Department of Electronics Engineering, Chungbuk National University, Cheongju, Korea; 5 Digestive Disease Center, Institute for Digestive Research, Department of Internal Medicine, Soonchunhyang University College of Medicine, Seoul, Korea; 6 Division of Gastroenterology and Hepatology, Department of Internal Medicine, Korea University College of Medicine, Seoul, Korea

**Keywords:** Video capsule endoscopy, Imaging and sensing

## Abstract

Three-dimensional (3D) reconstruction of capsule endoscopic images has been attempted for a long time to obtain more information on small bowel structures. Due to the limited hardware resources of capsule size and battery capacity, software approaches have been studied but have mainly exhibited inherent limitations. Recently, stereo camera-based capsule endoscopy, which can perform hardware-enabled 3D reconstruction, has been developed. We aimed to evaluate the feasibility of newly developed 3D capsule endoscopy in clinical practice. This study was a prospective, single-arm, feasibility study conducted at two university-affiliated hospitals in South Korea. Small bowel evaluation was performed using a newly developed 3D capsule endoscope for patients with obscure gastrointestinal bleeding, suspected or established Crohn’s disease, small bowel tumors, and abdominal pain of unknown origin. We assessed the technical limitations, performance, and safety of the new capsule endoscope. Thirty-one patients (20 men and 11 women; mean age: 44.5 years) were enrolled. There was no technical defect preventing adequate visualization of the small bowel. The overall completion rate was 77.4%, the detection rate was 64.5%, and there was no capsule retention. All capsule endoscopic procedures were completed uneventfully. In conclusion, newly developed 3D capsule endoscopy was safe and feasible, showing similar performance as conventional capsule endoscopy. Newly added features of 3D reconstruction and size measurement are expected to be useful in the characterization of subepithelial tumours.

## Introduction

Since its development in 2000, capsule endoscopy has played major roles in the evaluation of small bowel disease^1^. However, current capsule endoscopy has many limitations, and continuous research and development have been performed to overcome these limirations^2,3^. One of the major drawbacks is the inability of capsule endoscopy to measure the accurate size of the lesion. In addition, due to its passive movement and uncontrolled air insufflation, characterization of excavated or polypoid lesions is difficult in some cases (e.g., differentiation of mass and mucosal bulges)^4^. To solve these problems, the three-dimensional (3D) reconstruction of the capsule image has been attempted but has shown limited utility so far^4,5^. A typical 3D-imaging system requires multiple independent cameras to reconstruct three-dimensional structures. However, due to the limited hardware resources pertaining to capsule size and battery capacity, there has been no hardware-enabled 3D reconstruction to date. Instead, software approaches (e.g., Shape-form-Shading, SfS) have been used to reconstruct 3D small bowel structures^6,7^. Software approaches have the fundamental limitation of estimating accurate and robust 3D information without newly added image information. Even though this tool has shown some usefulness in the interpretation of capsule images, it has had limited clinical significance due to its inherent limitations^4,5^.

Recently, IntroMedic Company (Seoul, South Korea) has developed a new stereo camera-based capsule endoscope, which is the first hardware-enabled 3D capsule endoscopy device. This instrument can estimate the geometric structure of the small bowel, making it possible to reconstruct the 3D structure and accurately estimate the size of an object within a 10% relative error to the actual size^8^.

In this study, we aimed to evaluate the feasibility of this newly developed 3D capsule endoscope in clinical practice.

## Patients and Methods

### Study design and participants

This study was a prospective, single-arm, feasibility study conducted at two university-affiliated hospitals in South Korea (Dongguk University Ilsan Hospital and Kangwon National University Hospital). Small bowel evaluation was performed using MiroCam^®^ MC4000 (IntroMedic^®^ Co., Seoul, South Korea) capsule endoscopy. Inclusion and exclusion criteria were the same as those for the current application of capsule endoscopy. Capsule endoscopy was performed in patients with obscure gastrointestinal bleeding, suspected or established Crohn’s disease, small bowel tumours, and abdominal pain of unknown origin. Capsule endoscopy was not performed in patients in whom gastrointestinal obstruction or stenosis was suspected, nor was it performed in patients with swallowing disorders. The study was conducted in accordance with the guidelines of the Declaration of Helsinki and was approved by the Dongguk University Ilsan Hospital Institutional Review Board (IRB no. DUIH2019-06-001-001). For participants under the age of 18 years, informed consent was obtained from a parent.

### Capsule endoscopy device description

**Figure 1:**
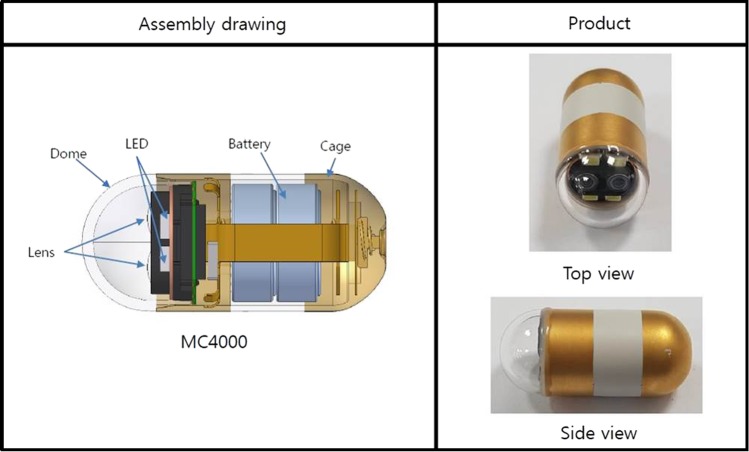
Assembly drawing of MC4000 (Image courtesy of IntroMedic^®^ Co., Seoul, South Korea).

MiroCam^®^ MC4000 is a newly developed stereo camera-based wireless capsule endoscope consisting of two cameras that are displaced by approximately 4 mm, four LED lights, a wireless transmitter, and a battery (Fig. 1)^8^. The size (10.8 mm*24.5 mm) and weight (3.4 g) are similar to those of previous versions of the MiroCam^®^ capsule endoscope, which are commercially available^9^. The depth range is up to 30 mm, the view angle is 170 degrees and the frame rate is 2*2 frames per second. For stereo matching, we computed the matching cost using the Census-based Hamming distance and absolute difference of intensities (AD-CENSUS)^10^. To consider the smoothness constraint with neighbouring pixels, we applied the SGM (Semi-Global Matching) to minimize the cost function with the constraint of the direct attenuation model^8,11^. With the aid of dual image sensors and a depth estimation algorithm, MC 4000 can render the 3D structure of the small bowel and estimate the size of the lesion within 10% relative error to the actual size, which has been demonstrated by a large bowel phantom model and plastic shaft phantom model^8,12^. Video clips showing the function of 3D reconstruction and size measurement are included (see Supplementary Videos S2 and S3).

### Capsule endoscopy procedures

Twelve hours of overnight fasting and bowel cleansing with purgatives (polyethylene glycol with ascorbic acid or oral sodium sulfate solution) were performed for all patients. Patients are allowed to drink clear fluids two hours after capsule ingestion. Written informed consent was obtained from all patients prior to the examination.

### Capsule endoscopy video evaluation

Captured images were evaluated by each hospital’s experienced reader (with more than 300 cases of capsule endoscopy performance). Images were analysed using the software MiroView (ver. 4.1, IntroMedic). Tens of thousands of images per patient were evaluated at a maximum speed of 10 frames per second in a single view mode and 20 frames per second in a double or quadruple view mode. Capsule image interpretation was performed according to the established guidelines^13–15^. If an abnormal finding was detected, the reader stopped displaying images and interpreted the lesion in the context of several images back and forth around the culprit image.

The quality of bowel preparation was categorized as follows: excellent, visualization of ≥75% of the mucosa; good, visualization of 50–74% of the mucosa; fair, visualization of 25–49% of the mucosa; poor, visualization of ≤24% of the mucosa. Excellent or good was considered adequate bowel preparation, and fair or poor was considered inadequate. Complete examination was defined as the capsule passing through the ileocaecal valve into the caecum during the recording time. Capsule retention was defined as the capsule remaining in the digestive tract for a minimum of two weeks or one that required directed intervention or therapy to aid its passage. The completion rate was calculated as the ratio of the successfully completed procedures to the total capsule endoscopy procedures. The retention rate was the ratio of the procedures with capsule retention to the total capsule endoscopy procedures. The detection rate was calculated as the ratio of the positive-detection procedures over the total capsule endoscopy procedures.

### Study endpoints

The primary objective of this study was to check the feasibility of the newly developed capsule in clinical practice. For this purpose, we assessed (1) the technical limitations and failures (problems associated with the functioning of the capsule) and (2) the performance and safety of the new capsule by analysing the detection, completion, and retention rate. The secondary objective was to check the usefulness of the 3D rendering and size measurement function in each patient’s clinical situation. We defined usefulness as any help in decision making on future management by additional information acquired through these two functions.

### Statistical analysis

This is a prospective, non-blinded, single-arm study to assess the feasibility of newly developed capsule endoscopy. No sample size calculation was performed. We estimated that 15 subjects would allow reasonable assessment of the general safety and feasibility of the newly developed capsule endoscope.

### Research involving human participants and Informed consent

All procedures performed in studies involving human participants were in accordance with the ethical standards of the institutional and/or national research committee and with the 1964 Helsinki Declaration and its later amendments or comparable ethical standards.

### Informed consent

Informed consent was obtained from all individual participants included in the study.

## Results

### Patients’ characteristics and bowel preparations

From June 2019 to January 2020, 31 patients (men 20, women 11; mean age 44.5, range 16–84) were enrolled in this study (Table 1). Indications for the study were obscure gastrointestinal bleeding, suspected Crohn’s disease, established Crohn’s disease, iron deficiency anemia, unexplained abdominal pain, suspected Behcet’s disease, and suspected juvenile polyposis (details are summarized in Supplementary Table S1). All patients received bowel preparation with purgatives, and most of them showed adequate quality of bowel preparation except eight patients with fair preparation and three patients with poor preparation (excellent 19.4%, good 45.2%, fair 25.8%, and poor 9.7%).

**Table 1:** Patients’ characteristics and bowel preparations.

Characteristics		Values
Age (range)		44.5 (16–84)
Sex (M:F)		20:11
Bowel preparation	Excellent	6 (19.4%)
	Good	14 (45.2%)
	Fair	8 (25.8%)
	Poor	3 (9.7%)

### Feasibility of the new capsule endoscopy

Clinically significant lesions were detected in 20 subjects, most of which were erosions and ulcers (detection rate 64.5%). The overall completion rate was 77.4%, and a complete examination was not possible for seven subjects. In two patients with Crohn’s disease, the capsule was retained in the small bowel near the ulcer and inflammation site for a long time but was excreted the next day. For the other patients, the capsule was retained around the suspicious small bowel mass in one patient, and the small bowel transit time was significantly prolonged in four patients (one of them had total gastrectomy for gastric cancer). However, there was no capsule retention (retention rate 0%). Moreover, there were no technical defects such as battery shortages, failure of capsule activation, and failure to download capsule images, which prevented adequate visualization of the small bowel (Table 2).

**Table 2:** Feasibility of the new capsule endoscopy.

Parameters	Values
Technical limitations or failures	0
Inability to swallow the capsule	0
Small bowel transit time (range)	362 min (206–577)
Incomplete small bowel examination	7 (22.6%)
Significant findings detected	20 (64.5%)
Capsule retention	0

### Capsule images and usefulness of the new device functions (3D rendering & size estimation)

**Figure 2:**
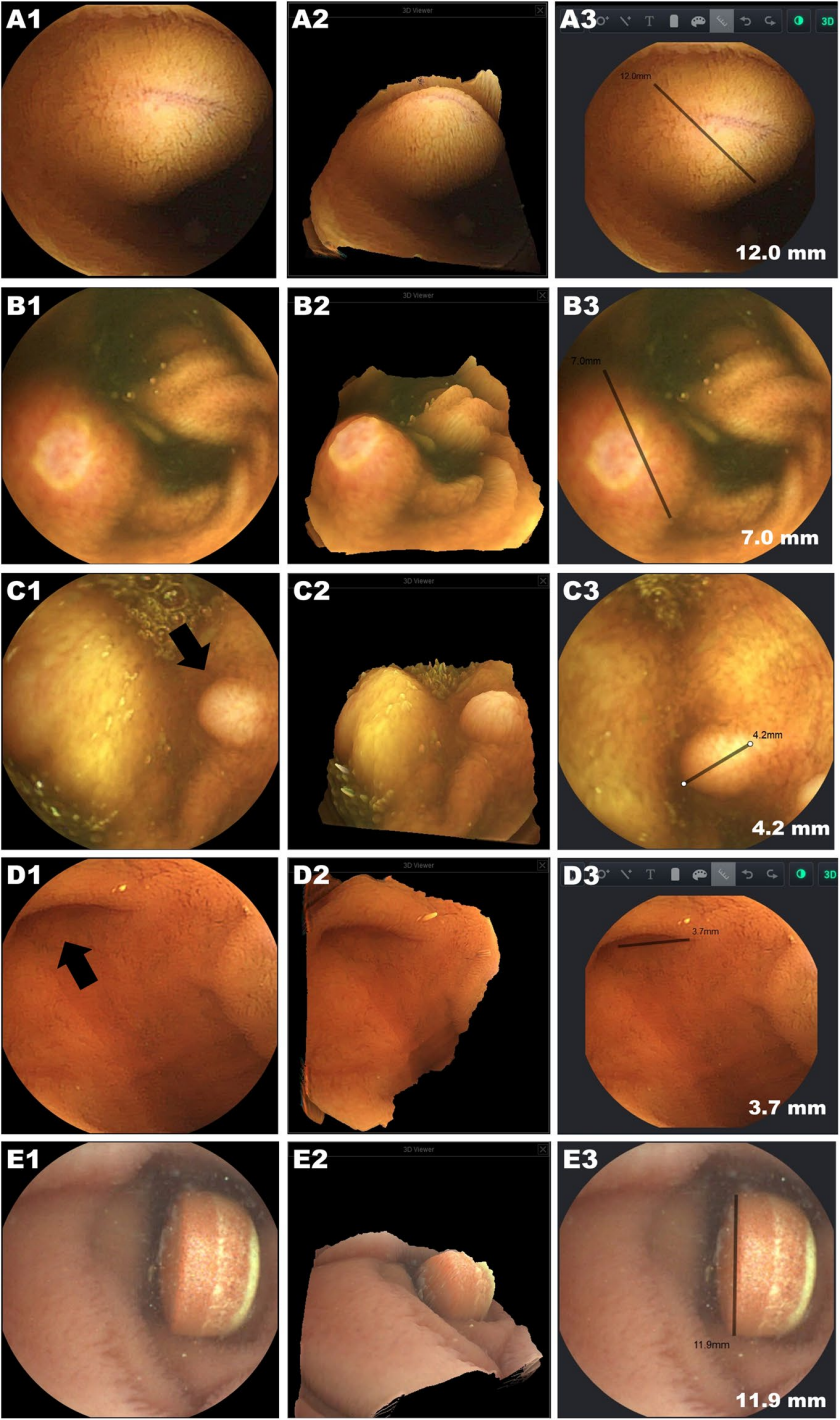
Examples of protruding or excavated structures detected by MC4000. A1, B1, C1, D1, and E1 are conventional 2D capsule images (A1: intraluminal protruding mass, B1: ulcer accompanied by mucosal swelling in a patient with Crohn’s disease, C1: small polyp, D1: diverticulum, and E1: oral pill encountered during capsule endoscopy). A2, B2, C2, D2, and E2 are the 3D reconstructions of the 2D image. A3, B3, C3, D3, and E3 show the size estimation function.

**Figure 3:**
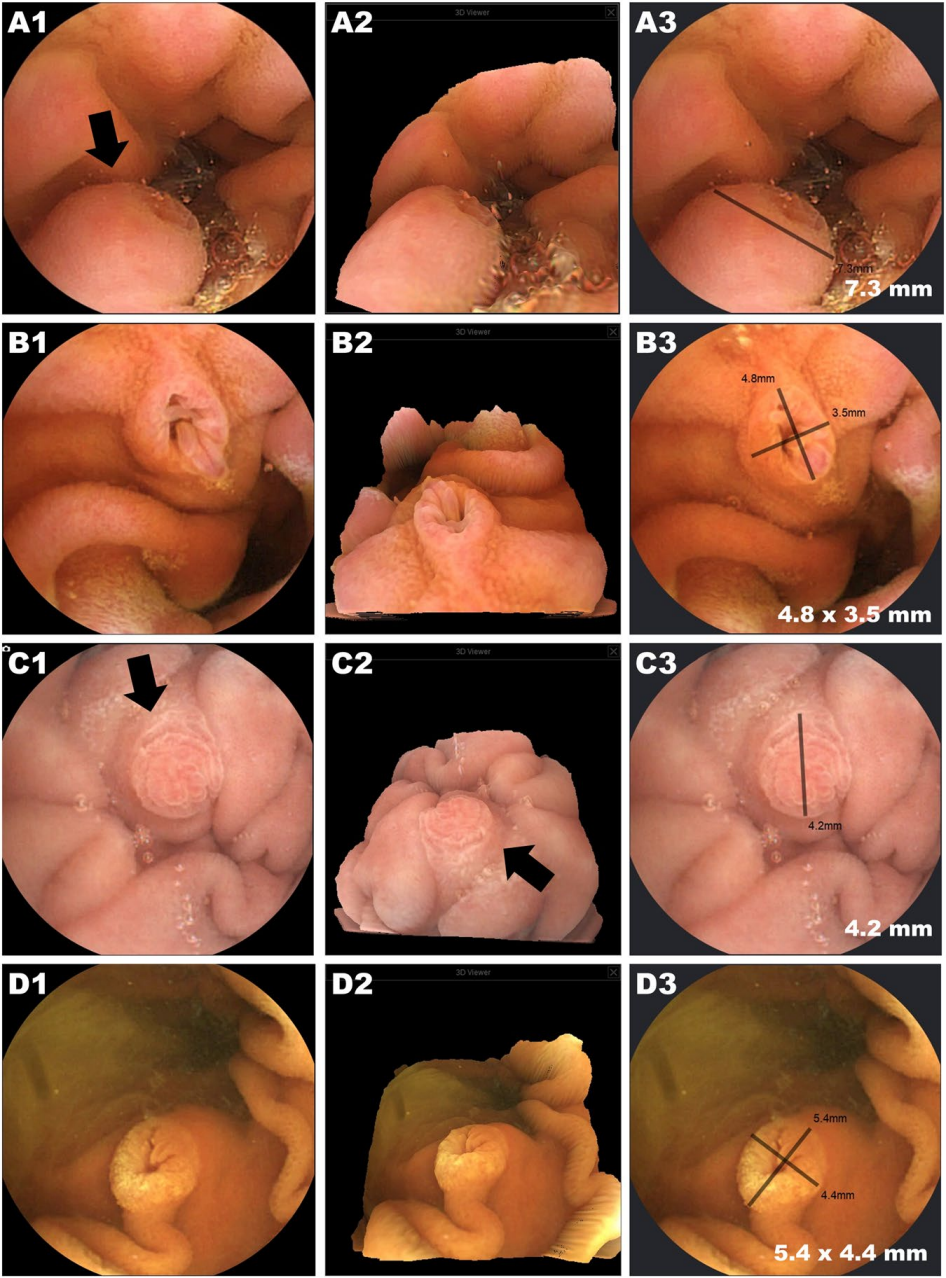
Examples of normal protruding structures (ampulla of Vater) detected by MC4000. A1, B1, C1, and D1 are conventional 2D capsule images showing the ampulla of Vater in different patients. A2, B2, C2, and D2 are the 3D reconstructions of the 2D image. A3, B3, C3, D3, and E3 show the size estimation function.

**Figure 4:**
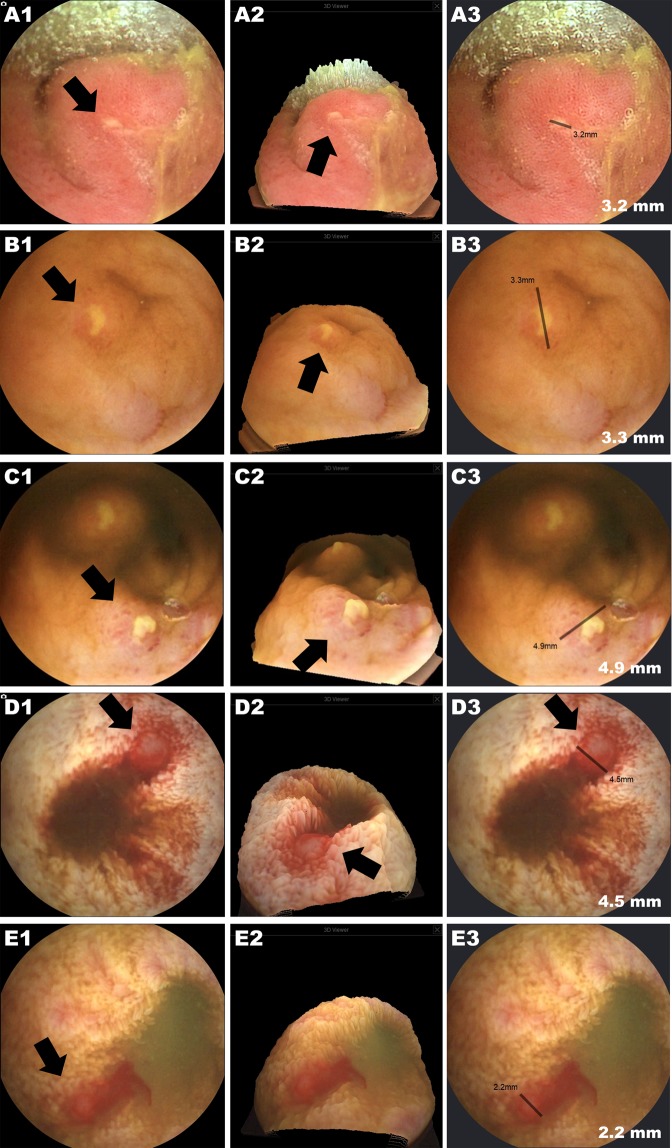
Examples of inflammatory lesions (erosions, ulcers, and bleeding) detected by MC4000. A1, B1, C1, D1, and E1 are conventional 2D capsule images (A1: erosion and edema of the mucosa, B1: aphthous ulcer, C1: two ulcers with an erythaematous mucosa, D1: deep ulcer with blood and clots, and E1: nodule with active bleeding in a patient with Crohn’s disease). A2, B2, C2, D2, and E2 are the 3D reconstructions of the 2D image. A3, B3, C3, D3, and E3 show the size estimation function.

Figures 2, 3, and 4 show lesions and other small bowel structures detected in this study. In a patient with a subepithelial lesion (Fig. 2A), 3D rendering and size estimation gave more information on the lesion characteristics compared to the conventional 2D capsule images and was helpful in discriminating the mass from an innocent mucosal bulge. In this patient, the capsule was retained around this mass for a long time, and he was recommended for further evaluation, including deep enteroscopy, but he declined and was lost during follow-up. For the other patients, most detected lesions were hyperaemia or erosion/small ulcers (Fig. 4). Even though 3D reconstruction with size measurement gave more information about the characteristics of these lesions, they did not contribute significantly to clinical decision making and future management.

## Discussion

Inability to control capsular movement is one of the major drawbacks of current capsule endoscopy. Due to this innate limitation, the characterization of small bowel lesions has been difficult in some cases (e.g., submucosal tumours), unlike conventional fibreoptic endoscopy, which can aerate, palpate, measure size, and acquire tissue. To obtain more information from limited image data from current capsule endoscopy, several efforts have been made, including three-dimensional reconstructions of the small bowel structure. There was a hardware approach using an infrared projector and a CMOS camera to construct a 3D structure, but the device failed commercialization due to size, power consumption, and packaging problems^6,16,17^. On the other hand, a software approach using the SfS technique yielded some promising results^6,7^. One study showed that 26.6% of 3D represented images made by this mathematical transformation presented enhanced visualization features compared to 2D conventional counterparts^5^. However, this function was helpful mainly for vascular lesions, which can be easily recognized and evaluated without the aid of 3D reconstruction. Image enhancement with 3D image representation software was not helpful for protruding lesions. In another study, the same image enhancement tool (SfS algorithm) was used to differentiate clinically significant masses from innocent mucosal bulges^4^. 3D reconstruction did not improve the performance of expert readers on the differentiation of masses from mucosal bulges, and there was limited use of this technique. Strictly speaking, there must be inherent limitations to the software approach. From the mathematical point of view, there is no information additional to that of the conventional 2D images, and only a change in visualization has been produced by the calculations of 2D image information. Therefore, previous studies showing the limitation of the software approach have consistently mentioned the need for hardware-enabled 3D reconstruction. Size estimation is also impossible by the software approach^4,5^.

In this study, we evaluated the feasibility of a newly developed stereo camera-based capsule endoscope, which enables the first hardware-enabled 3D capsule endoscopy. With the aid of dual image sensors and custom-developed algorithms, this device can objectively perform 3D reconstruction and size estimation of small bowel structures. We performed capsule endoscopy with this new device in 31 patients. There were no technical defects, including battery shortages, failure of capsule activation, and failure to download capsule images, and all capsule endoscopy procedures were uneventfully completed. Seven subjects showed incomplete examinations, and the overall study completion rate was 77.4%. The detection rate was 64.5%, and there was no capsule retention. This study showed values of the detection rate, completion rate and retention rate (64.5%, 77.4%, and 0%, respectively) that were comparable to those in previously published data with conventional capsule endoscopy (60–63%, 80–83%, and 1–2%, respectively)^18–22^. Even though the function of 3D reconstruction and size estimation was fascinating, these functions did not contribute significantly to the clinical management of most patients in this study. However, in one patient with a protruding small bowel mass, 3D reconstruction and size measurement added more information to characterize the lesion and were helpful in decision making.

Our feasibility study has several limitations. The small number of patients mostly consisting of subjects with inflammatory lesions was not enough to show the usefulness of the new function of capsule endoscopy. In addition, the accuracy of size measurement and the shape of the 3D rendered structure need to be confirmed in human data by surgery or deep enteroscopy. However, we were able to demonstrate the safety and technical feasibility of this newly developed capsule endoscope.

In conclusion, the newly developed 3D capsule endoscope MC4000 (IntroMedic® Co., Seoul, South Korea) was safe and feasible for small bowel evaluation and showed at least a similar performance as conventional capsule endoscopy. The clinical implications of the newly added fascinating features of 3D reconstruction and size measurement need to be validated in a larger number of patients. Particularly, subepithelial tumour characterization is expected to be the most promising part of the clinical applications of 3D capsule endoscopy.

## Supplementary information

Supplementary Video S2.

Supplementary Video S3.

Supplementary Table S1.
