# Supplementary material for: 3D reconstruction of small bowel lesions using stereo camera-based capsule endoscopy
Source: Sci Rep. 2020 Apr 7;10:6025. doi: 10.1038/s41598-020-62935-7 (PMC7138835; doi:10.1038/s41598-020-62935-7)
Supplement: Supplementary file 3 — Supplementary Table S1. [file 41598_2020_62935_MOESM3_ESM.pdf]

Title: 3D reconstruction of small bowel lesions using stereo camera-based capsule endoscopy

Seung-Joo Nam, M.D., Ph.D.[1], Yun Jeong Lim, M.D., Ph.D.*[2], Ji Hyung Nam, M.D., Ph.D.[2], Hyun Seok Lee, M.D., Ph.D.[3], Youngbae Hwang, Ph.D.[4], Junseok Park, M.D.[5], Hoon Jai Chun, M.D., Ph.D.[6]

[1]Department of Internal Medicine, Kangwon National University School of Medicine, Chuncheon, Korea

[2]Department of Internal Medicine, Dongguk University Ilsan Hospital, Goyang, Korea

[3]Department of Internal Medicine, School of Medicine, Kyungpook National University, Kyungpook National University Hospital, Daegu, Korea

[4]Department of Electronics Engineering, Chungbuk National University, Cheongju, Korea

[5]Digestive Disease Center, Institute for Digestive Research, Department of Internal Medicine, Soonchunhyang University College of Medicine, Seoul, Korea

[6]Department of Internal Medicine, Korea University College of Medicine, Seoul, Korea

Supplementary table S1. Patients' characteristics and findings of 3D capsule endoscopy

No.	Sex	Age	Indications	Bowel preparation	Complete examination	Capsule retention	Capsule diagnosis
1	M	22	OGIB	Excellent	Incomplete	No	Small bowel tumor
2	M	50	Suspicion of CD	Excellent	Complete	No	CD
3	M	56	OGIB	Excellent	Complete	No	NSAID enteropathy
4	F	62	Abdominal pain	Good	Complete	No	Foreign body
5	M	61	OGIB	Excellent	Complete	No	NSAID enteropathy
6	M	44	Established CD	Good	Complete	No	CD
7	M	46	Established CD	Good	Complete	No	CD
8	M	84	Anemia	Good	Incomplete	No	Non-specific enteritis
9	M	57	OGIB	Good	Complete	No	Non-specific enteritis
10	F	46	Suspicion of CD	Excellent	Complete	No	CD
11	F	33	Established CD	Good	Incomplete	No	CD
12	M	35	Suspicion of juvenile polyposis	Good	Complete	No	Normal
13	F	24	Established CD	Good	Complete	No	Intestinal parasite
14	M	28	Established CD	Excellent	Complete	No	CD
15	M	40	Established CD	Good	Incomplete	No	CD
16	M	40	Abdominal pain	Fair	Complete	No	Non-specific enteritis
17	M	43	Suspicion of CD	Fair	Complete	No	Non-specific enteritis
18	M	23	Established CD	Fair	Complete	No	CD
19	F	37	Anemia	Fair	Complete	No	Non-specific enteritis
20	M	53	Suspicion of CD	Fair	Complete	No	Non-specific enteritis
21	F	42	OGIB	Poor	Incomplete	No	Small bowel bleeding

22	M	49	Suspicion of Behcet’s disease	Fair	Complete	No	Behcet’s enteritis
23	F	16	Suspicion of CD	Good	Incomplete	No	CD
24	F	50	Suspicion of Behcet’s disease	Good	Complete	No	Non-specific enteritis
25	M	53	Abdominal pain	Good	Complete	No	Non-specific enteritis
26	F	57	OGIB	Fair	Complete	No	No evidence of small bowel bleeding
27	F	16	OGIB	Good	Complete	No	CD
28	M	43	Established CD	Poor	Complete	No	No definite small bowel lesions
29	M	68	OGIB	Poor	Incomplete	No	Non-specific enteritis
30	F	84	Anemia	Fair	Complete	No	No evidence of small bowel bleeding
31	M	17	Suspicion of CD	Good	Complete	No	Non-specific enteritis

OGIB: Obscure gastrointestinal bleeding, CD: Crohn’s disease, NSAID: Non-steroidal anti-inflammatory drugs
